# Supplementary material for: Reduction of Cardiovascular Events and Related Healthcare Expenditures through Achieving Population-Level Targets of Dietary Salt Intake in Japan: A Simulation Model Based on the National Health and Nutrition Survey
Source: Nutrients. 2022 Aug 31;14(17):3606. doi: 10.3390/nu14173606 (PMC9460310; doi:10.3390/nu14173606)
Supplement: Supplementary file 1 [file nutrients-14-03606-s001.zip › nutrients-1880989-supplementary.pdf]

Supplementary material

Reduction of cardiovascular events and related healthcare expenditures through achieving population-level targets of dietary salt intake in Japan: A simulation model based on the National Health and Nutrition Survey.

Nayu Ikeda, Hitomi Yamashita, Jun Hattori, Hiroki Kato, Katsushi Yoshita, Nobuo Nishi.

Correspondence: Nayu Ikeda, PhD
ikedan@nibiohn.go.jp
Section of Population Health Metrics
International Center for Nutrition and Information
National Institute of Health and Nutrition
National Institutes of Biomedical Innovation, Health and Nutrition
Tokyo, Japan

Table S1. Baseline data on total population, mean dietary salt intake, and mean systolic blood pressure by age group and sex in Japan, 2019

Sex, age (years)	Total population, thousand [1]	Mean dietary salt intake, g/day [2]	Mean systolic blood pressure, mmHg [2]
Men			
40–49	9,373	10.60	125.8
50–59	8,160	10.62	131.7
60–69	7,930	11.48	135.8
70–79	7,332	11.52	135.8
Women			
40–49	9,147	8.89	114.3
50–59	8,118	9.15	123.7
60–69	8,302	9.99	131.0
70–79	8,593	9.77	136.1

Table S2. Baseline data on the incidence, prevalence, and mortality of ischemic heart disease and stroke by age group and sex in Japan, 2019, based on estimates from the Global Burden of Disease Study [3]

Sex, age (years)	Incidence, per 100,000		Prevalence, per 100,000		Mortality, per 100,000		
	Ischemic heart disease	Stroke	Ischemic heart disease	Stroke	Ischemic heart disease	Stroke	All causes
Men							
40–49	134.7 (96.2, 180.3)	201.9 (161.5, 250.0)	862.1 (732.8, 1,017.7)	1,363.8 (1,139.2, 1,609.8)	16.6 (15.9, 17.2)	15.1 (14.2, 15.9)	150.6 (147.9, 153.4)
50–59	346.5 (247.5, 458.7)	363.0 (294.5, 439.8)	2,552.7 (2,191.9, 2,961.1)	3,055.7 (2,590.4, 3,593.4)	44.2 (42.6, 46.0)	35.2 (33.4, 37.1)	390.6 (383.1, 398.5)
60–69	609.3 (441.1, 791.2)	486.3 (386.7, 597.2)	6,008.3 (5,134.4, 7,022.8)	4,789.8 (4,152.9, 5,523.5)	98.9 (94.8, 102.8)	75.1 (71.2, 78.9)	996.9 (979.4, 1,015.4)
70–79	1,065.4 (781.2, 1,426.5)	607.9 (481.2, 758.3)	10,933.6 (9,402.7, 12,584.7)	7,190.4 (6,109.9, 8,328.9)	240.1 (221.8, 252.3)	212.0 (194.8, 224.9)	2,579.2 (2,538.1, 2,622.3)
Women							
40–49	27.8 (17.6, 41.4)	175.9 (136.7, 220.1)	478.4 (408.0, 568.0)	1,319.8 (1,112.4, 1,559.3)	3.5 (3.3, 3.7)	6.9 (6.4, 7.4)	88.2 (86.6, 90.0)
50–59	82.4 (56.0, 114.2)	353.1 (279.6, 438.4)	971.4 (834.9, 1,136.1)	3,141.2 (2,607.7, 3,734.0)	9.0 (8.4, 9.5)	14.8 (13.8, 15.7)	201.1 (197.4, 205.1)
60–69	227.4 (162.6, 301.2)	555.8 (437.3, 687.1)	2,300.7 (1,968.9, 2,715.7)	5,270.6 (4,476.6, 6,225.4)	27.1 (25.0, 28.7)	29.5 (27.2, 31.4)	439.3 (432.3, 446.8)
70–79	607.4 (445.4, 809.0)	745.6 (593.7, 913.8)	5,229.1 (4,436.4, 6,051.9)	7,320.6 (6,322.7, 8,492.5)	102.7 (85.8, 112.6)	98.6 (83.4, 107.8)	1,205.0 (1,187.4, 1,223.8)

Original estimates by 5-year age groups and sex in the Global Burden of Disease Study were averaged between two adjacent age groups to obtain estimates by 10-year age group and sex. Values in parentheses indicate lower and upper bounds of 95% confidence intervals.

Table S3. Baseline data on proportions of first-ever incident cases and acute case fatality of ischemic heart disease and stroke, by sex

Sex	Proportion of first-ever cases in incidence, %		28-day case fatality, % [4]	
	**Ischemic heart disease [5]**	**Stroke [6]**	**Ischemic heart disease**	**Stroke**
Men	92.4	71.8	34.3	14.9
Women	92.4	75.2	43.3	15.7

Table S4. Baseline data on epidemiologic associations used in the model, by age group, both sexes

Age (years)	Change in systolic blood pressure per 100 mmol/24-hour change in sodium intake [7]*	Relative risk per 10 mmHg increase of systolic blood pressure [3]†	
		**Ischemic heart disease**	**Stroke‡**
40–49	6.6 (4.6, 8.6)	1.568 (1.398, 1.799)	1.628 (1.354, 1.950)
50–59	9.2 (6.8, 11.6)	1.487 (1.385, 1.619)	1.521 (1.361, 1.698)
60–69	10.3 (7.8, 12.8)	1.405 (1.332, 1.488)	1.414 (1.302, 1.524)
70–79	10.3 (7.8, 12.8)	1.303 (1.225, 1.404)	1.284 (1.179, 1.389)

Values in parentheses indicate lower and upper bounds of 95% confidence intervals.
* Confidence intervals were calculated from point estimates and standard errors by authors. The value in the 60–69-year-old group were assigned to the 70–79-year-old group.
† Estimates in 5-year age groups of 40–44, 50–54, 60–64, and 70–74 years in the original data source were assigned to 40–49, 50–59, 60–69, and 70–79-year-old groups, respectively, in the simulation.
‡ Relative risks for stroke per 10 mmHg increase of systolic blood pressure were separated for ischemic and hemorrhagic strokes in the original data source. Relative risks for ischemic stroke were adopted to represent total stroke in the simulation, because mortality of ischemic stroke was higher than that of hemorrhagic stroke in Japan in 2019 [3].

Table S5. Baseline data on national healthcare expenditures for ischemic heart disease and stroke by age group and sex in Japan, 2019, in US dollars

Sex, age (years)	Outpatient [8]		Prescription drug [9] *		Inpatient [8]	
	**Ischemic heart disease**	**Stroke**	**Ischemic heart disease**	**Stroke**	**Ischemic heart disease**	**Stroke**
Men						
40–49	35,364,424 (152)	39,405,039 (158)	85,358,054	134,808,300	141,704,629 (7,533)	281,339,719 (7,800)
50–59	87,831,966 (152)	86,157,495 (148)	212,023,436	255,306,366	356,931,703 (7,600)	565,244,331 (7,328)
60–69	208,939,636 (156)	203,605,009 (143)	497,141,993	393,793,874	741,825,638 (7,523)	1,148,701,123 (6,846)
70–79	385,546,673 (165)	406,479,963 (147)	814,926,360	537,887,522	1,136,662,606 (7,336)	2,064,660,178 (6,539)
Women						
40–49	13,382,250 (141)	30,252,600 (163)	45,807,785	127,071,537	17,495,525 (5,973)	187,198,524 (8,677)
50–59	30,183,302 (142)	58,230,654 (145)	80,541,519	260,671,232	48,319,855 (5,911)	339,630,624 (8,055)
60–69	75,611,508 (141)	129,819,141 (137)	199,894,276	452,168,988	158,329,288 (6,496)	665,357,310 (7,507)
70–79	185,520,103 (142)	309,967,874 (137)	453,522,455	639,243,249	405,050,809 (6,552)	1,576,486,179 (6,876)

National healthcare expenditures on outpatient care were defined as the sum of expenditures on outpatient care and prescription drugs. Values in parentheses indicate healthcare expenditures per case calculated by authors.
* Data were available only for total cardiovascular diseases in the total population (approximately 7.9 billion US dollars) in the original data source. To obtain data on pharmaceutical drug costs for ischemic heart disease and stroke by age group and sex, the total cardiovascular drug cost was multiplied by proportions of people having a disease in each age group and sex in the total of people having these two diseases obtained from the Global Burden of Disease Study [3]. Pharmaceutical drug costs per case were therefore constant at approximately 1,072 US dollars for ischemic heart disease and stroke across sex-age groups.

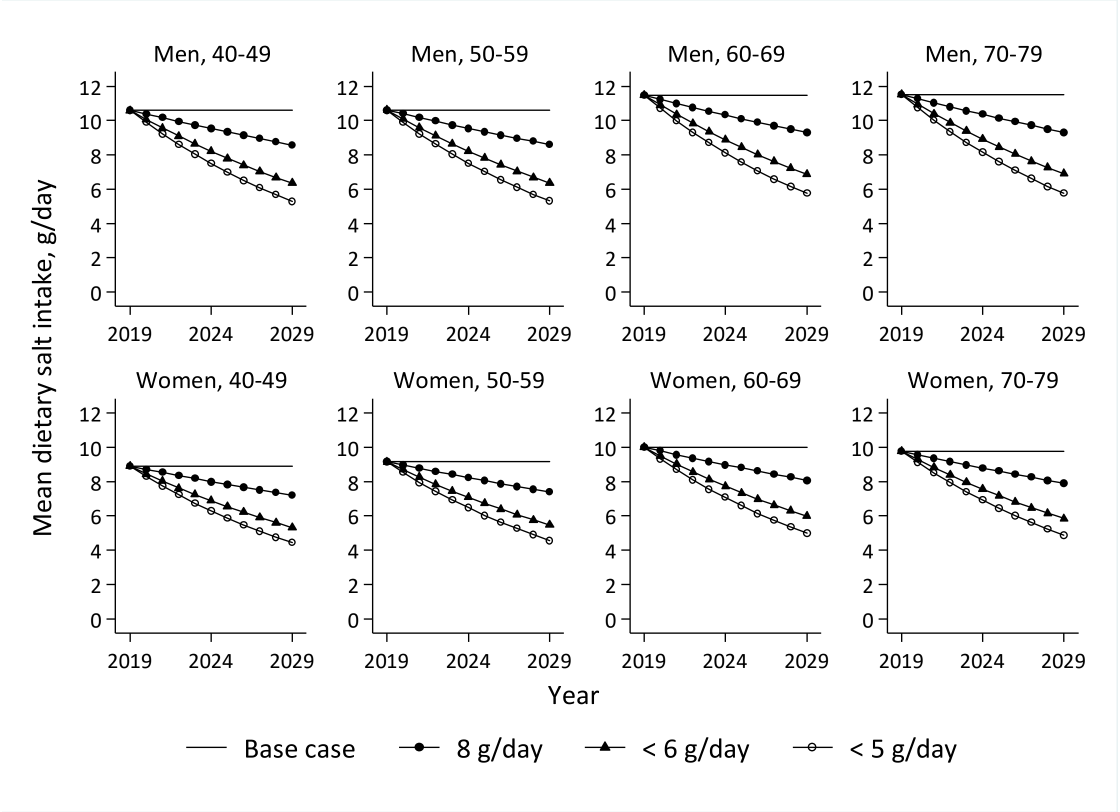

Figure S1. Mean dietary salt intake to be achieved to reach dietary salt reduction targets between the years 2019 and 2029, by age group and sex. Base case refers to sustaining the baseline levels of 2019; 8 g/day, to meet the Japanese national health promotion goal; < 6 g/day, per the Japanese Hypertension Society's Guidelines for the Management of Hypertension; and < 5 g/day, as specified by the World Health Organization.

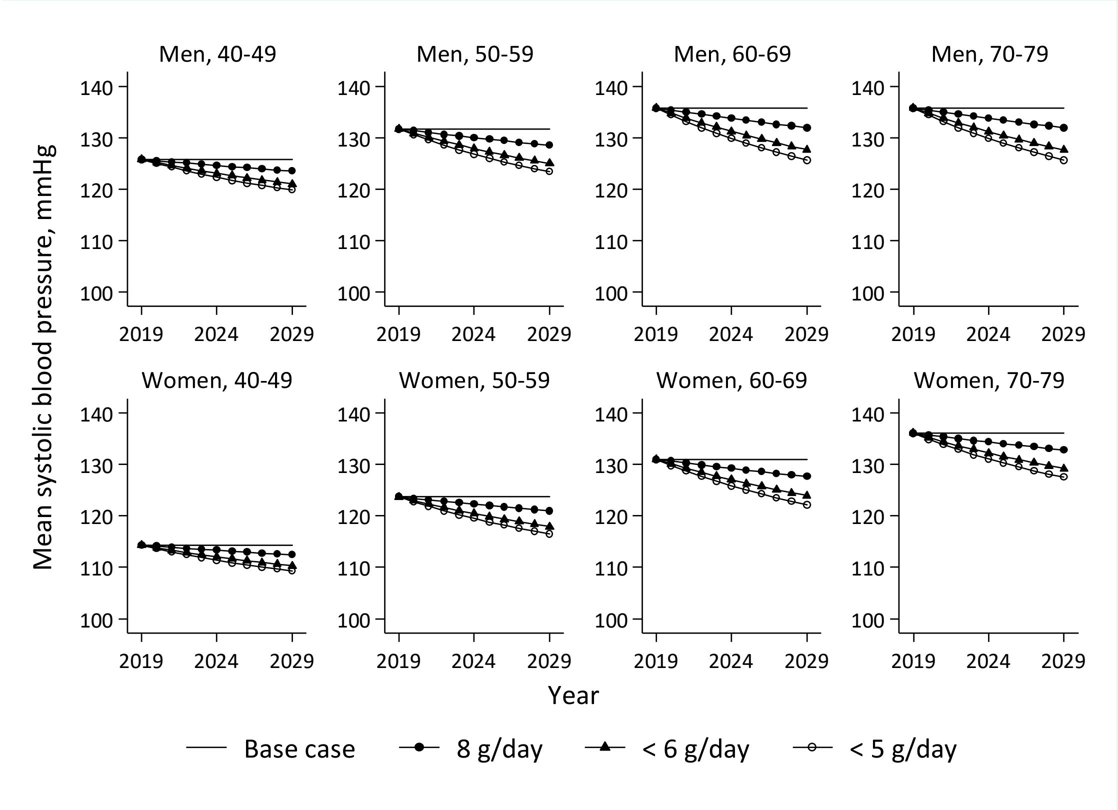

Figure S2. Mean systolic blood pressure to be achieved to reach dietary salt reduction targets between the years 2019 and 2029, by age group and sex. Base case refers to sustaining the baseline levels of 2019; 8 g/day, to meet the Japanese national health promotion goal; < 6 g/day, per the Japanese Hypertension Society's Guidelines for the Management of Hypertension; and < 5 g/day, as specified by the World Health Organization.

References

1. Statistics Bureau of Japan, Ministry of Internal Affairs and Communications. Current Population Estimates as of October 1, 2019. Available online: https://www.stat.go.jp/english/data/jinsui/2019np/index.html (accessed on 4 February 2022).
2. Ministry of Health, Labour and Welfare. *The National Health and Nutrition Survey in Japan, 2019*; Ministry of Health, Labour and Welfare: Tokyo, 2020.
3. Global Burden of Disease Collaborative Network. Global Burden of Disease Study 2019 (GBD 2019) Results. Available online: https://vizhub.healthdata.org/gbd-results/ (accessed on 25 July 2022).
4. Rumana, N.; Kita, Y.; Turin, T.C.; Nakamura, Y.; Takashima, N.; Ichikawa, M.; Sugihara, H.; Morita, Y.; Hirose, K.; Kawakami, K., et al. Acute case-fatality rates of stroke and acute myocardial infarction in a Japanese population: Takashima stroke and AMI registry, 1989-2005. *Int J Stroke* **2014**, *9 Suppl A100*, 69-75, doi:10.1111/ijs.12288.
5. Uchiyama, S.; Shibata, Y.; Hirabayashi, T.; Mihara, B.; Hamashige, N.; Kitagawa, K.; Goto, S.; Origasa, H.; Shimada, K.; Kobayashi, H., et al. Risk factor profiles of stroke, myocardial infarction, and atrial fibrillation: a Japanese Multicenter Cooperative Registry. *J Stroke Cerebrovasc Dis* **2010**, *19*, 190-197, doi:10.1016/j.jstrokecerebrovasdis.2009.04.004.
6. Takashima, N.; Arima, H.; Kita, Y.; Fujii, T.; Miyamatsu, N.; Komori, M.; Sugimoto, Y.; Nagata, S.; Miura, K.; Nozaki, K. Incidence, Management and Short-Term Outcome of Stroke in a General Population of 1.4 Million Japanese ― Shiga Stroke Registry ―. *Circulation Journal* **2017**, *81*, 1636-1646, doi:10.1253/circj.CJ-17-0177.
7. Law, M.R.; Frost, C.D.; Wald, N.J. By how much does dietary salt reduction lower blood pressure? I--Analysis of observational data among populations. *BMJ* **1991**, *302*, 811-815, doi:10.1136/bmj.302.6780.811.
8. Ministry of Health, Labour and Welfare. Overview of national medical care expenditure, 2019. Available online: https://www.mhlw.go.jp/toukei/saikin/hw/k-iryohi/19/index.html (accessed on 13 April 2022).
9. Ministry of Health, Labour and Welfare. Survey on Prescription Drug Expenditure, 2019. Available online: https://www.mhlw.go.jp/bunya/iryouhoken/database/zenpan/cyouzai_doukou.html (accessed on 10 February 2022).
